# P-527. A Scoping Review of HIV Pre-exposure Prophylaxis for Indigenous Peoples: Understanding Barriers and Facilitators to PrEP Access, Use and Adherence

**DOI:** 10.1093/ofid/ofae631.726

**Published:** 2025-01-29

**Authors:** Timothy Lim, Jaris Swidrovich

**Affiliations:** University of Toronto, Toronto, Ontario, Canada; University of Toronto, Toronto, Ontario, Canada

## Abstract

**Background:**

Indigenous Peoples in Canada, which include First Nations, Métis, and Inuit, experience disproportionately higher incidences of human immunodeficiency virus (HIV) infection compared to settler Canadians. Pre-Exposure Prophylaxis (PrEP) involves the daily use of antiretroviral drugs to prevent HIV transmission to HIV-negative people. When taken as prescribed, PrEP can reduce one's risk of getting HIV from sex by over 90% and by more than 70% among people who inject drugs. While PrEP awareness has increased over the years, reasons for its lack of uptake in Indigenous populations remains unknown. A scoping review was done to understand the current literature available how Indigenous Peoples in Canada, United States, Australia and New Zealand conceptualize PrEP.

**Methods:**

We searched electronic databases (Pubmed, Ovid Medline, i-Portal, and Native Health Databases) for studies published between January 2012 and December 2023 for articles using variants of the search terms “Pre-Exposure Prophylaxis” and “Indigenous” . Studies were included if they discussed barriers and/or facilitators to PrEP awareness, interest, uptake, or continuation among Indigenous Peoples. Studies were independently screened by two authors.

**Results:**

Results were synthesized narratively. Of 50 citations identified, 3 studies were eligible for inclusion. Four themes arose from the literature - all of which discussed barriers to PrEP: (1) limited Indigenous engagement and representation in research, (2) stigma and cultural barriers, (3) resource constraints and (4) geographical and socioeconomic barriers to PrEP.

**Conclusion:**

There is a paucity of literature exploring barriers and facilitators to PrEP access, use and adherence in Indigenous Peoples. Given the colonial history and violence that Indigenous Peoples faced and continue to face in western countries such as Canada, United States, Australia and New Zealand, greater attention to culturally safe and trauma informed interventions will likely improve PrEP awareness and use in Indigenous populations.

**Disclosures:**

**All Authors**: No reported disclosures

